# RETRACTION: Identification of Autophagy‐Related Genes in Diabetic Foot Ulcer Based on Bioinformatic Analysis

**DOI:** 10.1111/iwj.70601

**Published:** 2025-04-21

## Abstract

**RETRACTION:** LiD.‐L. , DingX.‐Y. , LongJ. , HeQ.‐L. , ZengQ.‐X. , LuN. , and ZouM.‐C. , “Identification of Autophagy‐Related Genes in Diabetic Foot Ulcer Based on Bioinformatic Analysis,” International Wound Journal 21, no. 3 (2024): e14476, 10.1111/iwj.14476.37909396 PMC10898398

The above article, published online on 01 November 2023, in Wiley Online Library (http://onlinelibrary.wiley.com/), has been retracted by agreement between the journal Editor in Chief, Professor Keith Harding; and John Wiley & Sons Ltd. Following an investigation by the publisher, all parties have concluded that this article was accepted solely on the basis of a compromised peer review process. The editors have therefore decided to retract the article. The authors disagree with the retraction.

**RETRACTION:** D.‐L. Li , X.‐Y. Ding , J. Long , Q.‐L. He , Q.‐X. Zeng , N. Lu , and M.‐C. Zou , “Identification of Autophagy‐Related Genes in Diabetic Foot Ulcer Based on Bioinformatic Analysis,” International Wound Journal 21, no. 3 (2024): e14476, 10.1111/iwj.14476.37909396 PMC10898398

The above article, published online on 01 November 2023, in Wiley Online Library (http://onlinelibrary.wiley.com/), has been retracted by agreement between the journal Editor in Chief, Professor Keith Harding; and John Wiley & Sons Ltd. Following an investigation by the publisher, all parties have concluded that this article was accepted solely on the basis of a compromised peer review process. The editors have therefore decided to retract the article. The authors disagree with the retraction.

